# Bioactive Compounds and Antioxidant Activity in the Fruit of Rosehip (*Rosa canina* L. and *Rosa rubiginosa* L.)

**DOI:** 10.3390/molecules28083544

**Published:** 2023-04-18

**Authors:** Fabiola Peña, Sebastián Valencia, Gonzalo Tereucán, Javiera Nahuelcura, Felipe Jiménez-Aspee, Pablo Cornejo, Antonieta Ruiz

**Affiliations:** 1 Departamento de Ciencias Químicas y Recursos Naturales, Scientific and Technological Bioresource Nucleus BIOREN-UFRO, Universidad de La Frontera, Avda. Francisco Salazar 01145, Temuco 4811230, Chile; 2 Programa de Doctorado en Ciencias Agroalimentarias y Medioambiente, Facultad de Ciencias Agropecuarias y Forestales, Universidad de La Frontera, Región de la Araucanía, Temuco 4811230, Chile; 3 Department of Food Biofunctionality (140b), Institute of Nutritional Sciences, University of Hohenheim, Garbenstr. 28, D-70599 Stuttgart, Germany; 4 Escuela de Agronomía, Facultad de Ciencias Agronómicas y de los Alimentos, Pontificia Universidad Católica de Valparaíso, Quillota 2260000, Chile

**Keywords:** anthocyanin, antioxidant activity, phenolic compounds, rosehip

## Abstract

Rosehips (*Rosa* spp., Rosaceae) are wild rose bushes with more than 100 species. Its fruits vary in colour and size, depending on the species, and are recognised for their nutritional characteristics. Ten samples of *Rosa canina* L. and *Rosa rubiginosa* L. fruits were collected at different geographical points from Southern Chile. Nutrients such as crude protein and minerals and functional properties such as phenolic compounds, ascorbic acid, and also antioxidant activities were evaluated by HPLC-DAD-ESI-MS/MS. The results revealed a high content of bioactive compounds, primarily ascorbic acid (6.0 to 8.2 mg g^−1^ fresh weight (FW)), flavonols (427.9 ± 0.4 μg g^−1^ FW) and antioxidant activity. We established a relationship between the antioxidant activity using Trolox equivalent antioxidant capacity (TEAC), cupric reducing antioxidant capacity (CUPRAC) and 2,2-diphenyl radical (DPPH) methods and the concentration of uncoloured compounds, such as flavonols and catechin. This antioxidant activity was primarily associated with the samples from Gorbea, Lonquimay, Loncoche, and Villarrica localities, and all of them were of the species *Rosa rubiginosa* L. The results here obtained represent novel information of rosehip fruits. In this sense, the reported information about compounds and antioxidant activities in rosehip fruits allowed us to continue new lines of research in relation to the potential formulation of new functional foods and also in the treatment and/or prevention of some diseases.

## 1. Introduction

The functional food characteristics of berries have been widely studied due to their high content of phenolic compounds, including flavonoids, such as anthocyanins, flavonols, flavan-3-ols, phenolic acids and hydrolysable tannins [1]. These compounds, individually or in combination, are primarily responsible for the health benefits of berries and associated with their antioxidant properties [2]. Some of these constituents are bioaccessible and bioavailable and exert anticancer, antimicrobial and anti-inflammatory activities and other activities [3]. Numerous in vitro studies indicate that plant secondary metabolites may affect various processes in mammalian cells, including gene expression, apoptosis, low-density lipoprotein oxidation, intracellular signaling, P-glycoprotein activation and the modulation of enzymatic activities associated with carcinogen activation and detoxification [4]. There is a wide variety of species from various botanical families called berry (berries in plural), which contain high levels of bioactive compounds that provide multiple health benefits [5]. Rosehip (*Rosa* spp.) fruits have gained attention for their important role in the health and cosmetic industries. According to the INFOR newsletter, rosehip fruit is considered a non-timber forest product (NTFP) with an important use in the cosmetic, pharmaceutical and gastronomy industries. In addition, it is positioned as the main NTFP in Chile with 30.7% of total exports, with 4420 tons of rosehip fruit, reaching USD 25.1 million, exceeding the export records in 2020. The main buyer countries include Germany, United States and Sweden. In addition, vegetable oil (7.7%) and rosehip seeds (1.3%) are also exported with amounts that reach USD 6.3 million and USD 1 million, respectively [6]. The genus Rosa has its origins in Eastern Europe [7] and includes more than 100 species. Three Rosa species are present in Chile: *R. rubiginosa* L., *R. canina* L. and *R. moschata* Herrm. [8]. Rosehip plants are perennial shrubs with an average height of approximately 2–3 m and strong resistance to harsh environmental conditions, such as rocky, sloping sites, poor soils and limited water access [9]. Fruits are characterised by lengths between 14.0 and 28.8 mm, diameters between 13 and 20 mm and weights of 1.2 to 2.7 g. The pulp of the fruit contributes to 42.9% to 66.5% of the fruit weight, depending on the species [10]. Various minerals, such as phosphorus, potassium, calcium, magnesium, iron, zinc, copper and manganese, have been reported in rosehip fruit [11]. Rosehip fruits have the highest content of vitamin C among fruits and vegetables (935–1230 mg 100 g^−1^) [10]. The fruits also contain important levels of vitamins A, B1, B2, B6, D, E and K [11,12]. The organic profile includes malic, quinic, tartaric and citric acids [13]. Fruits have a total phenolic content between 290 and 1385 mg 100 g^−1^ [12,13,14,15], and catechin and procyanidin B2 are the main phenolic compounds [16]. Carotenoids, such as lycopene, β-cryptoxanthin, β-carotene, rubixanthin, gazaniaxanthin and zeaxanthin, have also been identified [17,18]. High antioxidant activity has been reported for the genus [19], which is associated with the presence of flavonoids, tannins, terpenoids, xantonoids and glycerol glycoside, but significant variability exists between rose species [20,21]. Previous studies compared different species of rosehip and showed similar qualitative profiles but important differences in the content of bioactive compounds in the fruit [20,22,23]. The highest polyphenol content was reported for *Rosa canina* [20]. In this sense, it is important to carry out a nutritional analysis which allows knowing information such as proximal composition (moisture, ashes, protein, fat and crude fiber) and mineral content, as well as functional analysis that allows us to determine antioxidant activity, compounds phenolics and vitamins [24].

Based on these findings and the high content of functional compounds in rosehip, we hypothesized that there would be a difference in the content and concentrations of bioactive compounds in rosehip fruits, which would be influenced by the species and the geographical area of collection. The present study determined the profiles and concentrations of bioactive compounds and antioxidant activities of two species of rosehip fruits.

## 2. Results

### 2.1. Protein and Nutrient Composition

The protein content (Table 1) in the rosehip fruits ranged from 0.69 to 1.10 mg g^−1^, without significant differences between species or locations. The magnesium contents (0.75 to 1.45 mg g^−1^) also showed no significant differences between the samples and collections evaluated. Significant differences were observed for calcium, phosphorus and potassium between locations, independent of the species collected (Table 1). For calcium, the highest content was in the sample from Carahue (18.13 ± 0.25 mg g^−1^), and the lowest value was found in the sample from Osorno (1.95 ± 0.00 mg g^−1^). For phosphorus, the highest value was detected in the sample from Lonquimay (6.04 ± 0.27 mg g^−1^), and the lowest concentration was detected in the sample from Gorbea (0.81 ± 0.02 mg g^−1^). For potassium, the highest value was observed in the sample collected in Melipeuco (8.75 ± 0.12 mg g^−1^), and the lowest content was found in the sample collected in Loncoche (2.93 ± 0.06 mg g^−1^).

### 2.2. Profiles and Concentrations of Phenolic Compounds and Ascorbic Acid in Rosehip

Phenolic compound identification was performed in rosehip fruits. Only nine phenolic compounds were tentatively identified according to their fragmentation patterns using mass spectrometry (Table 2). Of the identified compounds, one anthocyanin (cyanidin-3-glucoside), one flavan-3-ol (catechin), one hydroxycinnamic acid (galloylquinic acid) and six flavonols (primarily glycosyl derivatives of quercetin) were identified by their UV profiles and fragmentation patterns (Table 2). The identification of catechin and cyanidin-3-glucoside was confirmed via comparison to commercial standards.

**Figure 1:**
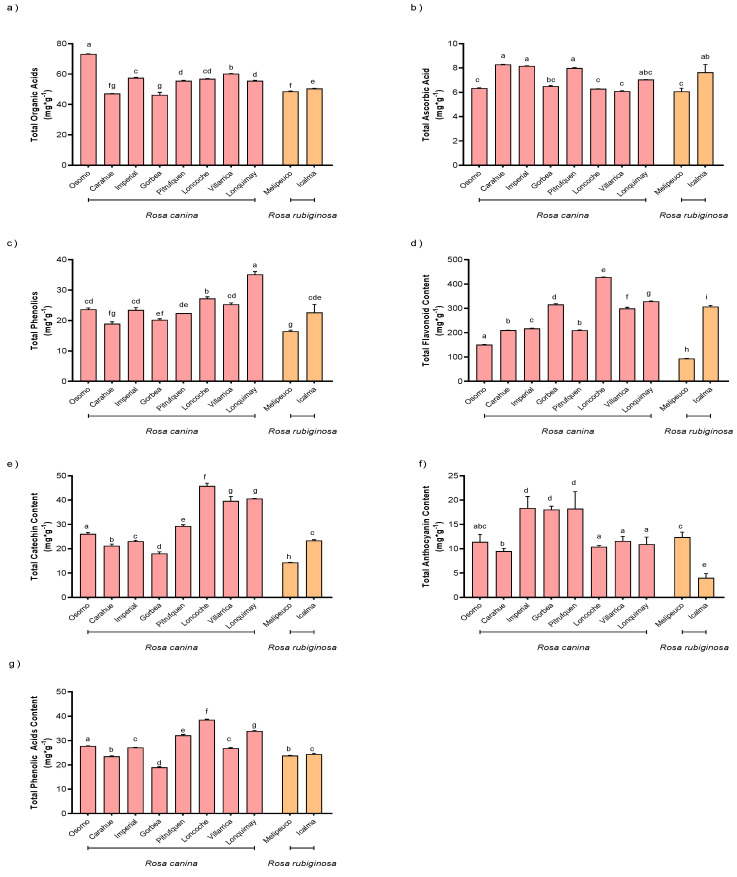
Contents in fruits of *Rosa* spp.: (**a**) organic acids; (**b**) ascorbic acid; (**c**) total phenolic; (**d**) flavonoid; (**e**) catechin; (**f**) anthocyanin; (**g**) phenolic acid content. Concentrations were expressed as fresh weight (FW). Different letters in each sub-figure indicate the presence of statistically significant differences according to the Tukey’s multiple range test (*p* ≤ 0.05; *n* = 9).

Individual phenolic compound concentrations were determined using HPLC-DAD (Table S1). The organic acid content ranged from 46.2 ± 1.66 mg g^−1^ to 73.2 ± 0.03 mg g^−1^ fresh weight (FW) (Figure 1a). The ascorbic acid concentration ranged from 6.0 to 8.2 mg g^−1^ FW. The lowest concentration was found in the samples collected in Melipeuco, and the highest concentration was found in the samples collected in Carahue (Figure 1b). Total phenolic content determination was consistent with the results of individual compounds. The lowest value was detected in the samples collected in Melipeuco, and the maximum value was found in the samples collected in Lonquimay (Figure 1c). The concentrations of flavonols were visibly higher and reached values of 93.6 ± 0.8 μg g^−1^ and 427.9 ± 0.4 μg g^−1^ FW in the samples collected in Melipeuco and Loncoche, respectively (Figure 1d). The contents of catechin were 14.3 ± 0.09 mg g^−1^ to 45.7 ± 1.15 mg g^−1^ FW for the samples collected in Melipeuco and Loncoche, respectively (Figure 1e). Total anthocyanin concentrations were 10.0 ± 0.0 μg g^−1^ and 40.1 ± 0.1 μg g^−1^ FW for the samples collected in Melipeuco and Pitrufquen, respectively (Figure 1f). Phenolic acid concentrations showed the same order of magnitude as anthocyanins and reached values up to 38.5 ± 0.2 mg g^−1^ FW in the samples collected in Loncoche (Figure 1g). A significant difference was observed between the two species collected, where *R. canina* had the lowest values compared to anthocyanins, flavonols, flavan-3-ols and total phenols. For the other compounds, no significant differences were observed between the two species.

### 2.3. Colour Parameters

The present study evaluated the compositions of red, yellow, and blue colourations, colour intensity and tonality (Table 3). The highest percentage of yellow colouration was detected in the *R. canina* samples collected in Icalma and Melipeuco, with 78.9% ± 2.2% and 71.8% ± 7.2%, respectively. For *Rosa rubiginosa*, a higher percentage of red colouration was found for all of the samples, and the highest values were found in the samples collected in Pitrufquén (35.92% ± 2.62%) and Osorno (35.06% ± 1.33%). This observation is consistent with the clear difference in the appearance of these species. *R. canina* tended to exhibit yellow colouration, and *R. rubiginosa* tended to have a red colouration. The intensity of the colour of the samples ranged from 0.26 ± 0.40 to 0.97 ± 0.01, and that of the sample collected in Loncoche showed the highest value. Tonality values ranged from 0.96 ± 0.11 to 3.97 ± 0.49, and the sample collected in Imperial showed the highest tonality value.

### 2.4. Antioxidant Activity in Rosehip Fruits

**Figure 2:**
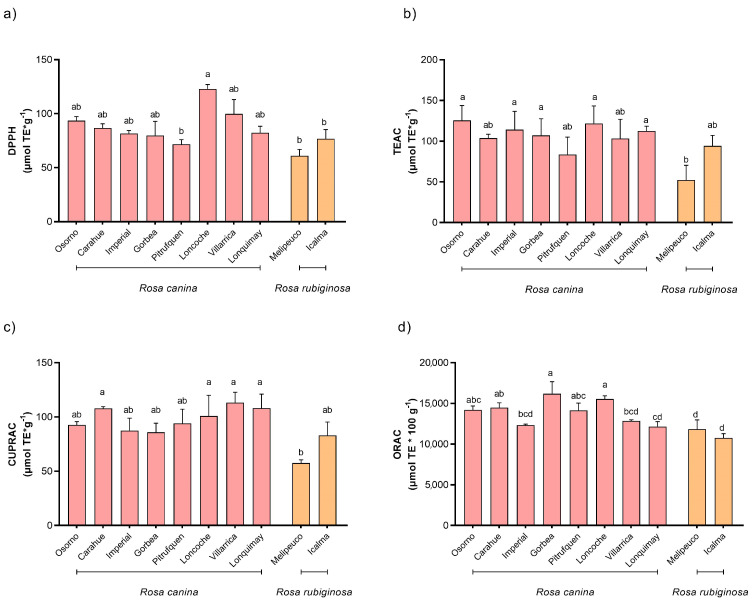
Antioxidant activity by spectrophotometric methods: (**a**) 2,2-diphenyl radical method (DPPH); (**b**) Trolox equivalent antioxidant capacity (TEAC); (**c**) cupric reducing antioxidant capacity (CUPRAC); (**d**) Oxygen Radical Antioxidant Capacity (ORAC). Concentrations were expressed as fresh weight (FW). Different letters in each sub-figure indicate the presence of statistically significant differences according to the Tukey’s multiple range test (*p* ≤ 0.05; *n* = 9).

The antioxidant activity of rosehips was determined using the TEAC, DPPH, CUPRAC and ORAC methods. The DPPH method showed that the fruits collected in Loncoche had the highest levels of antioxidant activity, with concentrations of 122.8 ± 4.2 μmol g^−1^ (Figure 2a). The TEAC method showed that the sample with the highest value was collected in Osorno (125.5 ± 18.3 μmol g^−1^) (Figure 2b). The CUPRAC method found the highest antioxidant activity in the sample collected in Villarrica (113.3 ± 9.5 μmol g^−1^) (Figure 2c). Consistent with the quantitative composition of the fruit, the lowest values for the DPPH, TEAC and CUPRAC methods were found in the sample collected in Melipeuco. The values obtained from the ORAC method (Figure 2d) ranged from 10,776.8 ± 509.7 to 16,190.6 ± 1487.3 µmol 100 g^−1^, which corresponded to the samples collected in Icalma and Gorbea, respectively. The highest results in all of the methodologies used corresponded to those of *R. rubiginosa* samples, and the lowest values corresponded to those of *R. canina* samples.

## 3. Discussion

The mineral content detected was higher than those in previous reports for rosehips and other common fruits. The mineral contents of rosehip fruits have been reported for phosphorus (0.61 mg g^−1^), potassium (4.29 mg g^−1^), calcium (1.69 mg g^−1^), magnesium (0.69 mg g^−1^), iron (1.06 mg g^−1^), zinc (0.25 mg^−1^), copper (0.113 mg^−1^) and manganese (1.02 mg^−1^) [11]. Compared to other berries, the mineral content in rosehip is also higher. For example, raspberries have lower contents of calcium (0.25 mg g^−1^), phosphorus (0.29 mg g^−1^), potassium (0.15 mg mg^−1^) and magnesium (0.22 mg g^−1^) [25]. Blueberries contain various minerals, such as magnesium (1.5–3.3 mg 100 g^−1^), potassium (2.7–4.4 mg 100 g^−1^), phosphorus (6.0–13.3 mg 100 g^−1^) and calcium (0.9–2.2 mg 100 g^−1^) [26].

The (poly)phenolic composition of rosehip fruits has been reported. It been reported that cyanidin-3-glycoside is the only anthocyanin present in seedless extracts, and 21 other compounds, including glycosides of quercetin, taxifolin, eriodyctiol and the dihydrochalcone phoridzin, were also reported [27]. The reported phenolic compounds in crude extracts of *R. canina* fruits include glycosylated flavonols, flavonol aglycones, a stilbenoid, four flavan-3-ols and cyanidin-3-glycoside as anthocyanin. Fetni et al. [21] identified 46 phenolic compounds in *R. canina* rosehips from Slovenia, including cyanidin-3-glycoside as anthocyanin, flavan-3-ols and proanthocyanidins as the most abundant group of compounds, simple phenolic acids, flavanons, flavonols, flavanones and the dihydrochalcone phloridzin. Fetni et al. [21] recently described over 25 different compounds in *R. canina* fruits collected in Algeria, including O-glycosylated flavanones, O-methylated flavones, O-glycosylated flavonols and aglycones of flavanonols, but they did not report any anthocyanin.

The results for anthocyanins are consistent with the literature. Only one anthocyanin has been detected in rosehip fruits, cyanidin-3-glucoside (cy-3-glu) [20,28]. The content of cy-3-glu ranges from 9.2 to 125.7 µg g^−1^ expressed per dry weight (DW) [13]. The abundance and content of anthocyanins in rosehips are lower compared to those of other commonly consumed fruits [29]. The values of phenolic compounds obtained in the present research are significantly lower than in other studies, which reached the maximum of 118.56 mg g^−1^ [14]. Several phenolic acids and their derivatives have been identified in rosehips. The most abundant are gallic and ellagic acids [13], with concentrations between 0.18 mg g^−1^ and 2.44 mg g^−1^ DW, which are notably lower than in our study. Catechin is the main flavonoid in all Rosa spp. species. Our results are significantly higher than those reported by Elmastaş et al. [23], with values in the range of 0.225 mg g^−1^ to 0.472 mg g^−1^. Cunja et al. [22] reported values of total organic acids higher than 260 mg g^−1^ FW, and the higher contents of citric acid and malic acid are notable. Organic acids are fundamental to the texture and flavour of fruit [30].

Our values for the content of total phenols are comparable with those reported by other authors, where the highest and lowest levels of total phenolic compounds in rosehips were 52.94 mg g^−1^ and 31.08 mg g^−1^, respectively [17,20]. We found a high content of total phenols in rosehip fruit (67.84 mg g^−1^ DW), which was similar to the study of Murathan et al. [31]. It is important to consider that the Folin–Ciocalteu method is not specific for phenolic compounds, but also responds to other organic compounds, such as sugars, proteins or ascorbic acid, giving an overestimation of the phenolic content [32]. In addition, lipophilic and hydrophilic antioxidants, such as tocopherols, retinol, carotenoids, ascorbate and phenols [33], can also be identified by the Folin–Ciocalteau method.

Rosehip fruits are recognised for their high content of ascorbic acid, and our results are comparable to the literature. The ascorbic acid content in rosehip fruit ranges from 1.80 to 10.9 mg g^−1^ FW [19,20,34,35]. The fruit of the rosehip has a higher content of ascorbic acid compared to other fruits, such as oranges (0.44 mg g^−1^) [36], blueberries (0.73 mg g^−1^) [37,38] and strawberries (0.56 mg g^−1^) [39].

Colour is an important attribute of fruits that affects the consumer perception of attractiveness and quality. Phenolic compounds, carotenoids, chlorophylls and betalains normally contribute to the food colour characteristics of fruits [40]. Rosehip fruits vary in colouration, and fruits present with yellow, red and orange pigmentation, which is primarily determined by the genotype and its state of maturity [13]. The orange to red colour of rose hips is caused by their carotenoid composition, primarily β-carotene and lycopene [41]. The presence of anthocyanins in flowers, fruits and vegetables is associated with their orange, red and blue colours [42]. Fascella et al. [20] and Cunja et al. [13] observed significant differences in colour parameters between wild and cultivated rosehip fruits.

Analyses of the antioxidant capacity of a plant or fruit are essential from a nutraceutical perspective, and rosehip fruits are valuable sources of bioactive compounds, including antioxidants [20]. Some studies reported that the antioxidant activity of extracts correlated with total, rather than individual, phenolic compounds of rosehip fruit [43]. This correlation is consistent with our results, where the antioxidant activities determined by DPPH TEAC and CUPRAC were directly related to total phenols and primarily represented by flavonols and flavan-3-ols. Our results of the antioxidant activity of rosehips are consistent with the literature. Roman et al. [19] found that the antioxidant activity of rosehip extracts measured using the DPPH method yielded values of 63.35 μM Trolox 100 g^−1^. Another study used an ORAC assay to determine the antioxidant capacity of rosehip extracts and found that it ranged between 2727 and 4577 μmol TE g^−1^ DW [20]. Compared to other fruits, rosehips exhibit higher antioxidant capacity. On the other hand, a content of 30 ± 4 mmol TE 100 g ^−1^ has been reported in ripe rosehip fruit according to the ORAC method, and a content of 9.1 ± 1.0 mmol TE 100 g^−1^ was measured using the TEAC method [35]. The antioxidant activity of raspberries (*Rubus idaeus* L.) has been reported as 29.0 μmol TE g^−1^ DW using the DPPH method [44]. Values from 50.8 to 99.5 μmol g^−1^ were determined in calafate (*Berberis microphylla*) using the TEAC method [45]. Values ranged from 2627 to 6747 μmol 100 g^−1^ DW in blueberry (*Vaccinium* spp), when the analysis was ORAC [46]. Oyarzún et al. [47] determined the antioxidant activity of chaura (*Gaultheria* sp.) fruits using the TEAC, DPPH and CUPRAC methods and obtained the maximum results of 18.72 μmol g^−1^, 10.22 μmol g^−1^ and 113.41 μmol g^−1^, respectively.

Fascella et al. [20] evaluated phenolic compounds and antioxidant activities in four species (*R. canina*, *Rosa corymbifera*, *Rosa micrantha* and *Rosa sempervirens*). However, a difference may be established from our results due to the species studied, because *R. canina* and *R. sempervirens* showed the highest antioxidant activities and the highest amounts of bioactive compounds. High antioxidant activity and a high content of phenolic compounds, especially flavonols, have been determined. This compound is highly important, because its biological effects are associated with its antioxidant activity related to its chemical structure [48]. Antioxidant activity is an important factor in inhibiting or delaying the oxidation of susceptible cell substrates [46]. Anti-inflammatory properties have also been reported in rosehip fruit [49]. Therefore, it is possible to use the bioactive compounds present in rosehip fruit as potential functional compounds for food processing.

**Figure 3:**
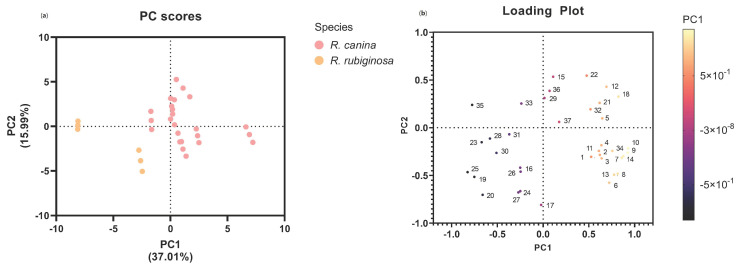
Principal component analysis (PCA) results: (**a**) the score plot generated by the two principal components indicating the difference between species; (**b**) the loading plot showing the variables that contribute to each one of the two principal components. The nomenclature of the varibles are as follows: (1) total phenolic content; (2) TEAC; (3) DPPH; (4) CUPRAC; (5) hydroxycinnamic acid 1; (6) flavonol 1; (7) flavonol 2; (8) flavonol 3; (9) flavonol 4; (10) flavonol 6; (11) flavonol 7; (12) anthocyanin 1; (13) total flavonoid content; (14) catechin; (15) total anthocyanins; (16) total protein content; (17) nitrogen content; (18) intensity of colour; (19) tone; (20) %yellow; (21)%red; (22)%blue; (23) a parameter; (24) b parameter; (25) L parameter; (26) C parameter; (27) h parameter; (28) potassium content; (29) calcium content; (30) magnesium content; (31) phosphorus content; (32) ORAC; (33) organic acid 1; (34) organic acid 2; (35) organic acid 3; (36) ascorbic acid content; (37) total organic acids.

The principal component analysis (PCA) of the studied variables in the present study showed the formation of homogeneous groups of experimental variables. PC1 explained 36.2% of the total experimental variance, and PC2 explained 15.3% of the total experimental variance (Figure 3). The PCA showed a relationship between the antioxidant activity determined by DPPH, TEAC, CUPRAC and the concentrations of uncoloured compounds, such as flavonols and catechin, which were reported for other fruits [47]. This antioxidant activity was primarily associated with the samples from Gorbea, Lonquimay, Loncoche and Villarrica and all of the species *R. rubiginosa*. The antioxidant activity determined by ORAC was related to the presence of anthocyanins, which provided the colour intensity of primarily red.

Finally, although rosehip fruits contain higher concentrations of bioactive compounds from the phenolic compound family and also ascorbic acid that possess an important antioxidant activity, there is scarce information about the benefits for human health in relation to their consumption. In this sense, it is necessary to carry out new studies about their potential beneficial properties. On the other hand, in Chile, rosehip fruits are not cultivated in large areas, limiting their consumption to local recollection.

## 4. Materials and Methods

### 4.1. Sample Collection

Rosehip fruit samples were collected in Southern Chile between April and June 2021 in 10 different locations: Carahue (Lat. 38°43′2.38″ S, long. 73°10′6.83″ W, alt. 38,334 m a.s.l.) Nueva Imperial (Lat. 38°42′37.95″ S, long. 73°1′13.28″ W, alt. 39,964 m a.s.l.), Pitrufquén (Lat. 39°0′8.399″ S, long. 72°45′41.91″ W, alt. 90,050 m a.s.l.), Gorbea (Lat. 39°8′20.20″ S, long. 72°38′11″ W, alt. 89. 412 m a.s.l.), Loncoche (Lat. 39°19′46.36″ S, long. 72°32′14.08″ W, alt. 100,528 m a.s.l.), Villarrica (Lat. 39°16′6.35″ S, long. 72°13′49.22″ W, alt. 224,976 m a.s.l.), Osorno (Lat. 40°33′10.9″ S, long. 73°12′18.3″ W, alt. 23,524 m a.s.l.), Lonquimay (Lat. 8°50′50″ S, long. 71°40′36″ W, alt. 1160,662 m a.s.l.), Melipeuco (Lat. 38°51′8.2″ S, long. 71°45′25.2″ W, alt. 912,664 m a.s.l.) and Icalma (Lat. 38°48′00″ S, long. 71°17′00″ W, alt. 1160 m a.s.l.). Carahue and Nueva Imperial are located on the coast, whereas Lonquimay, Melipeuco and Icalma are located in mountain ranges. The species collected in Melipeuco and Icalma were identified as *Rosa canina* L. and *Rosa rubiginosa* L. for the other collection areas (Figure S1). The samples were stored at −80 °C until processing.

### 4.2. Protein and Mineral Determinations

For the total protein determination, 1 g of sample was placed in a digestion tube. Kjeldahl catalyst and 10 mL of 95–98% sulfuric acid were added in the tube. Then, digestion was carried out at 400 °C for 30 min. Then, 50 mL of water, 25 mL of boric acid and 2–3 drops of a mixed indicator were added, and the solution was distilled for 10 min. Finally, the distillate was titled with HCl (0.31 N), until the solution turned green or violet. The crude protein content was determined using the Kjeldahl method based on the quantification of organic nitrogen [50]. 

Regarding mineral determinations, for extract preparation, 1 g of sample was dried at 70 °C. Then, it was maintained in a muffle for 4–8 h at 500 °C. One millilitre of water and 10 mL of HCl 2 M were added and heated until boiling. Finally, the residues were filtered and resuspended in water for the analyses. Potassium, calcium and magnesium contents were determined after acid digestion using atomic absorption spectrophotometry (AAS; Unicam SOLAAR, mod. 969, Cambridge, UK). The phosphorus content was determined using the ammonium molybdate reaction to form molybdene blue under acidic conditions, which was quantified at 655 nm [51].

### 4.3. Determination of Phenolic Compounds Using HPLC

The extraction and chromatographic separation processes were performed as previously described [52]. For the preparation of the extract, 1 g of fruit was homogenised with 2 mL of an extraction solvent (90% methanol, 9% water and 1% formic acid) and sonicated for 1 min at a 40% amplitude. Subsequently, it was shaken for 10 min. Then, it was centrifuged for 10 min, and the supernatant was removed and stored in the dark. High-performance liquid chromatography-diode array detection (HPLC-DAD) analyses were performed using an HPLC system (Shimadzu, Tokyo, Japan) equipped with a quaternary LC-20AT pump, a DGU-20A5R degassing unit, a CTO-20A oven, an SIL-20a autosampler and a UV-visible diode array spectrophotometer SPD-M20A. Instrument control and data collection were performed using Lab Solutions software (version 5.96) (Shimadzu, Duisburg, Germany). The chromatographic analyses were performed according to Ruiz et al. [53]. Identity assignments were performed using an HPLC-DAD system coupled to a 6545-quadrupole time-of-flight (Q-ToF) mass spectrometer (Agilent, Waldbronn, Germany). The control software used here was a Mass Hunter workstation (version B.06.11).

Phenolic compounds were quantified using external calibration at the maximum wavelength of each compound family (520 nm for anthocyanins, 360 nm for flavonols, 320 nm for hydroxycinnamic acid and 280 nm for flavan-3-ols) using cyanidin-3-glucoside, quercetin, chlorogenic acid and catechin as standards, respectively. Total phenolic content was estimated using the Folin–Ciocalteu method (Table S2) [52].

### 4.4. Determination of Organic Acids

Low-molecular-weight organic acids (LMWOAs) were determined in rosehip fruit using HPLC-DAD. Briefly, 0.5 g of fruit was homogenised in 5 mL of 0.2 mol L^−^^1^ CaCl_2_, shaken for 10 min and centrifuged for 10 min at 4000× *g*. Chromatographic analyses were performed using a C_18_ Symmetry Waters column (250 × 4.6 mm, 5 μm) with a Novapak Waters C_18_ precolumn (22 × 3.9 mm, 4 μm) set at 30 °C. The chromatographic run was performed for 15 min using phosphoric acid (0.2 N, pH 2.1) as the mobile phase at 1.0 mL min^−^^1^. Quantification was performed at 210 nm via external calibration using citric acid as a standard (Table S2) [52].

### 4.5. Ascorbic Acid Content

The ascorbic acid content was determined using HPLC-DAD. Briefly, 5 g of fresh fruit was crushed in 25 mL of 100 mg L^−^^1^ oxalic acid and subjected to ultrasound at a 20% amplitude for 45 s, followed by centrifugation for 20 min at 4000× *g*. The supernatant was filtered and injected into an HPLC-DAD system. Chromatographic analysis was performed using a Zorbax Eclipse Agilent C_18_ column (250 × 4.6 mm, 5 µm) and a Novapak Waters C_18_ precolumn (22 × 3.9 mm, 4 µm) at 40 °C. Liquid chromatography was performed using an isocratic method with 2% formic acid in water (A) and 2% formic acid in acetonitrile (B) as the mobile phase at 0.7 mL min^−^^1^ at 40 °C. Quantification was performed at 254 nm via external calibration [52].

### 4.6. Colour Parameters

Colour determinations were performed using the CieLAB method, in a 1 mm quartz cuvette in a Genesys 10s UV–Vis spectrophotometer (Thermo Scientific, Waltham, MA, USA) [54].

### 4.7. Determination of Antioxidant Activity

The antioxidant activity of rosehip fruits was determined using three colourimetric methods: Trolox equivalent antioxidant capacity (TEAC), cupric reducing antioxidant capacity (CUPRAC) and the 2,2-diphenyl radical (DPPH) method [52]. The fluorimetric method was performed to estimate the oxygen radical absorbance capacity (ORAC) as reported by Ou et al. [55]. Measurements were performed in a microplate reader (SYNERGY HTX, BioTek Instruments, Winooski, VT, USA).

### 4.8. Statistical Analysis

For all of the studied variables, a hierarchical ANOVA was performed, where the 2 rosehip species were nested into the 10 localities considered. The significance level was established at *p* ≤ 0.05. The means were compared by Tukey’s multiple range test. Datasets were also subjected to principal component analysis (PCA) and correlation analyses to establish the relationships between different variables.

## 5. Conclusions

The present results revealed a high content of bioactive compounds, such as ascorbic acid and phenolic compounds, and related antioxidant activities in rosehip fruits. We established a relationship between phenolic compounds and their antioxidant activities, and flavonols were the most abundant compounds in the rosehip fruit. Anthocyanins were responsible for colouration. We did not establish a marked difference in the bioactive compounds between the studied species, because a difference was observed only in the content of anthocyanins and antioxidant activity using the ORAC method. No trend was observed for the other analysed parameters between the species.

The higher concentrations of bioactive compounds and the higher levels of antioxidant activity are of great interest and could contribute to the potential development of new subproducts or functional foods or in preventive or therapeutic use in some disorders and pathologies. In this sense, it is necessary to carry out new studies where the aforementioned nutraceutical characteristics of the fruits would be in use.

## Figures and Tables

**Table 1:** Determination of protein by the Kjeldahl method, minerals (Mg, Ca and K) by atomic absorption spectrophotometry and phosphorus by a colorimetric method in rosehip fruits. Different letters in each sub-figure indicate the presence of statistically significant differences according to the Tukey’s multiple range test (*p* ≤ 0.05; *n* = 9).

Location	Proteins (mg g^−1^)	Ca (mg g^−1^)	Mg (mg g^−1^)	P (mg g^−1^)	K (mg g^−1^)
Carahue	0.70 ± 0.21 ^a^	18.13 ± 0.25 ^a^	0.78 ± 0.05 ^a^	0.90 ± 0.04 ^f^	4.55 ± 0.07 ^g^
Gorbea	1.01 ± 0.20 ^a^	4.87 ± 0.08 ^b^	0.79 ± 0.23 ^a^	0.81± 0.02 ^f^	6.97 ± 0.28 ^c^
Icalma	1.10 ± 0.33 ^a^	2.43 ± 0.01 ^f^	0.97 ± 0.35 ^a^	1.53 ± 0.15 ^e^	4.46 ± 0.10 ^g^
Imperial	0.77 ± 0.15 ^a^	2.93 ± 0.03 ^e^	0.81 ± 0.05 ^a^	2.11 ± 0.06 ^d^	4.92 ± 0.05 ^f^
Loncoche	0.69 ± 0.07 ^a^	2.99 ± 0.03 ^e^	0.75 ± 0.16 ^a^	0.82 ± 0.04 ^f^	2.93 ± 0.06 ^h^
Lonquimay	0.97 ± 0.20 ^a^	3.06 ± 0.03 ^e^	0.92 ± 0.12 ^a^	6.04 ± 0.27 ^a^	8.09 ± 0.08 ^b^
Melipeuco	0.99 ± 0.11 ^a^	3.68 ± 0.001 ^d^	1.45 ± 0.43 ^a^	4.60 ± 0.07 ^b^	8.75 ± 0.12 ^a^
Osorno	0.94 ± 0.27 ^a^	1.95 ± 0.00 ^g^	0.75 ± 0.04 ^a^	4.17 ± 0.08 ^c^	6.51 ± 0.11 ^d^
Pitrufquén	1.08 ± 0.19 ^a^	4.02 ± 0.01 ^c^	0.77 ± 0.53 ^a^	1.56 ± 0.03 ^e^	4.76 ± 0.17 ^g^
Villarica	1.08 ± 0.10 ^a^	2.46 ± 0.03 ^f^	0.76 ± 0.02 ^a^	1.97 ± 0.11 ^d^	6.02 ± 0.08 ^e^

**Table 2:** Identification of phenolic compounds in rosehip fruits by HPLC-DAD-ESI-MS/MS.

Peak Number	t_R_ (min)	Abbreviation	Tentative Identification	[M]+	[M-H]^−^	Product Ions	٨_max_ (nm)
1	5.1	CAT1	Catechin	-	289.1	-	279
2	10.1	ANT1	Cyanidin-3-glucoside	449.1	-	287.1	516
3	15.3	HCAD1	Galloylquinic acid	-	343.1	191.0; 205.0; 111.0	280
4	16.9	FLAV1	n.i	-	449.1	269.1; 152.0	289
5	17.5	FLAV2	n.i	-	433.1	271.1	278
6	18.5	FLAV3	n.i	-	615.1	465.10; 301.0	355
7	19.0	FLAV4	Quercetin-hexoside	-	461.1	300.0	353
8	19.8	FLAV6	Quercetin-glucuronide	-	477.1	300.0	353
9	20.9	FLAV7	Quercetin-rhamnoside	-	447.1	300.0	353

Note: n.i means no identified.

**Table 3:** Colour parameters including colour intensity, hue, yellow, red, blue, a, b, L, C and h, by CieLAB method in fruit of rosehip from different locations. Different letters in each sub-figure indicate the presence of statistically significant differences according to the Tukey’s multiple range test (*p* ≤ 0.05; *n* = 9).

Location	Colour Intensity	Tonality	% Yellow	% Red	% Blue	a	B	C	L	h
***Rosa rubiginosa* L.**										
Carahue	0.72 ± 0.10 ^b^	1.47 ± 0.20 ^c^	51.27 ± 5.43 ^cd^	34.90 ± 1.07 ^b^	13.83 ± 4.58 ^bcd^	−4410.03 ± 510.28 ^de^	16.63 ± 4.36 ^ab^	3.44 ± 2.12 ^b^	81.78 ± 2.36 ^b^	62.59 ± 12.00 ^b^
Gorbea	0.57 ± 0.06 ^b^	1.27 ± 0.17 ^c^	53.49 ± 3.72 ^cd^	42.49 ± 3.13 ^a^	4.02 ± 02.03 ^cde^	−3655.55 ± 123.05 ^bcd^	15.54 ± 1.31 ^b^	10.91 ± 2.00 ^a^	83.95 ± 1.60 ^b^	52.84 ± 5.85 ^b^
Imperial	0.76 ± 0.7 ^b^	1.60 ± 0.17 ^bc^	46.47 ± 2.12 ^de^	29.12 ± 1.94 ^c^	24.41 ± 1.04 ^ab^	−4277.55 ± 541.50 ^de^	12.26 ± 3.11 ^b^	−5.90 ± 1.98 ^c^	82.21 ± 1.15 ^b^	56.92 ± 11.08 ^b^
Loncoche	0.97 ± 0.01 ^a^	1.28 ± 0.08 ^c^	42.45 ± 1.19 ^de^	33.15 ± 1.25 ^bc^	24.40 ± 0.73 ^ab^	−5006.74 ± 208.24 ^e^	10.64 ± 1.70 ^b^	−5.44 ± 1.33 ^c^	76.93 ± 0.09 ^c^	50.92 ± 6.09 ^b^
Lonquimay	0.73 ± 0.08 ^b^	1.41 ± 0.09 ^c^	46.65 ± 0.86 ^de^	33.30 ± 2.55 ^bc^	20.06 ± 3.16 ^abc^	−4122.90 ± 423.66 ^bcd^	12.23 ± 1.31 ^b^	−0.82 ± 3.35 ^bc^	81.97 ± 1.95 ^b^	52.39 ± 3.07 ^b^
Osorno	0.58 ± 0.12 ^b^	1.57 ± 0. 24 ^bc^	54.71 ± 6.28 ^cd^	35.06 ± 1.33 ^b^	10.22 ± 5.00 ^cde^	−3787.05 ± 105.49 ^bcd^	16.02 ± 2.55 ^b^	4.95 ± 1.28 ^ab^	84.77 ± 1.74 ^b^	59.64 ± 7.14 ^b^
Pitrufquen	0.75 ± 0.03 ^b^	0.96 ± 0.11 ^c^	34.34 ± 1.30 ^de^	35.92 ± 2.62 ^b^	29.74 ± 1.45 ^a^	−3119.37 ± 133.29 ^abc^	−0.57 ± 1.15 ^c^	−4.96 ± 2.01 ^c^	81.69 ± 1.03 ^b^	15.46 ± 5.37 ^c^
Villarrica	0.61 ± 0.12 ^b^	2.15 ± 0.25 ^b^	62.28 ± 5.89 ^bc^	28.99 ± 0.70 ^c^	8.73 ± 5.23 ^cde^	−4521.25 ± 535.90 ^de^	22.91 ± 1.34 ^a^	2.89 ± 1.88 ^b^	84.73 ± 2.49 ^b^	83.21 ± 4.54 ^a^
***Rosa canina* L.**										
Melipeuco	0.34 ± 0.03 ^c^	3.97 ± 0.49 ^a^	71.78 ± 7.28 ^ab^	18.18 ± 2.29 ^d^	10.04 ± 8.36 ^cde^	−2893.11 ± 206.27 ^ab^	16.53 ± 0.80 ^b^	−0.21 ± 3.26 ^bc^	91.44 ± 0.87 ^a^	66.33 ± 1.98 ^ab^
Icalma	0.26 ± 0.4 ^c^	3.66 ± 0.08 ^a^	78.90 ± 2.20 ^a^	21.57 ± 1.09 ^d^	−0.47 ± 3.29 ^e^	−2485.68 ± 321.96 ^a^	15.71 ± 1.75 ^b^	3.40 ± 0.74 ^b^	92.58 ± 0.76 ^a^	60.70 ± 6.09 ^b^

## Data Availability

The data presented in this study are available on request from the corresponding author.

## Supplementary Materials

The following supporting information can be downloaded at: https://www.mdpi.com/article/10.3390/molecules28083544/s1, Table S1: Concentrations of individual phenolic compounds in fruit of *Rosa* spp. by HPLC-DAD and their antioxidant activities by spectrometry methods. Abbreviations: HCAD1—hydroxycinnamic acid; FLAV1—flavonol 1; FLAV2—flavonol 2; FLAV3—flavonol 3; FLAV4—flavonol 4; FLAV6—flavonol 6; FLAV7—flavonol 7; ANT1—anthocyanin all with the same unit of measurement (µg g^−1^). Table S2: Analytical parameters for HPLC and spectrophotometric methods. Abbreviations: DL—detection limit; QL—quantification limit; LR—linear range; CV%—coefficient of variation; TEAC—Trolox equivalent antioxidant capacity; CUPRAC—cupric reducing antioxidant capacity; DPPH—2,2-diphenyl radical methods; ORAC—oxygen radical absorbance capacity. Figure S1: (**A**) *Rosa rubiginosa*; (**B**) *Rosa canina* fruits.

## Sample Availability

Samples of the compounds are available from the authors.
