# Supplementary material for: Bioactive Compounds and Antioxidant Activity in the Fruit of Rosehip (Rosa canina L. and Rosa rubiginosa L.)
Source: Molecules. 2023 Apr 18;28(8):3544. doi: 10.3390/molecules28083544 (PMC10144129; doi:10.3390/molecules28083544)

Table S1: Concentration of individual phenolic compounds in fruit of Rosa spp. by HPLC-DAD and antioxidant activity by spectrometry methods. Where: HCAD1: hydroxycinnamic acid, FLAV1: flavonol 1, FLAV2: flavonol 2, FLAV3: flavonol 3, FLAV4: flavonol 4, FLAV6: flavonol 6, FLAV7: flavonol 7, ANT1: anthocyanin all with the same unit of measurement (ug g^{-1}).

Sample	HCAD1 μg g^{-1}	FLAV1 μg g^{-1}	FLAV2 μg g^{-1}	FLAV3 μg g^{-1}	FLAV4 μg g^{-1}	FLAV6 μg g^{-1}	FLAV7 μg g^{-1}	ANT1 μg g^{-1}
**Carahue**	23.5 ± 0.3g	63.7 ± 0.3f	36.8 ± 0.3d	69.5 ± 0.1d	9.9 ± 0.1d	17.9 ± 0.1e	12.2 ± 0.4g	37.3 ± 0.0b
**Gorbea**	19.0 ± 0.2h	131.1 ± 2.6b	52.1 ± 1.0b	81.3 ± 0.4c	11.3 ± 0.7c	25.1 ± 1.1b	15.0 ± 0.1e	29.7 ± 0.2e
**Imperial**	27.1 ± 0.1de	72.7 ± 0.5e	31.8 ± 0.1e	71.0 ± 0.5d	10.2 ± 0.3d	17.8 ± 0.1e	13.6 ± 0.2f	23.9 ± 0.2g
**Loncoche**	38.6 ± 0.2a	169.4 ± 2.5a	67.8 ± 1.8a	116.1 ± 0.6a	16.3 ± 0.1a	34.6 ± 0.2a	23.9 ± 0.2b	31.8 ± 0.2d
**Lonquimay**	33.9 ± 0.2b	120.8 ± 0.9c	43.9 ± 0.2c	97.4 ± 0.2b	14.6 ± 0.4b	21.0 ± 0.2d	30.9 ± 0.4a	25.2 ± 0.0f
**Osorno**	27.8 ± 0.0d	53.1 ± 0.2g	23.4 ± 0.3f	44.8 ± 0.5f	6.7 ± 0.1f	11.0 ± 0.0g	11.8 ± 0.3g	19.8 ±0.2h
**Pitrufquen**	32.2 ± 0.4c	70.5 ± 0.8e	33.7 ± 1.2e	61.1 ± 0.5e	8.9 ± 0.1e	16.4 ± 0.3f	18.9 ± 0.3c	40.2 ± 0.2a
**Villarrica**	26.9 ± 0.2e	114.1 ± 2.7d	50.1 ± 1.9b	84.2 ± 1.8c	11.3 ± 0.4c	22.6 ± 0.4c	17.2 ± 0.2d	33.9 ± 0.3c
**Melipeuco**	23.8 ± 0.2fg	26.4 ± 0.1h	16.0 ± 0.2f	30.6 ± 0.3g	3.1 ± 0.1g	3.3 ± 0.1h	14.2 ± 0.1ef	10.0 ± 0.1j
**Icalma**	24.4 ± 0.4g	134.6 ± 1.5b	31.4 ± 0.9e	97.7 ± 2.4b	9.5 ± 0.2de	15.3 ± 0.5f	18.7 ± 0.7c	11.1 ± 0.1i

Table S2: Analytical parameters for HPLC and spectrophotometric methods. Where DL: detection limit, QL: quantification limit, LR: linear range, CV% coefficient of variation, TEAC: Trolox equivalent antioxidant capacity, CUPRAC: cupric reducing antioxidant capacity, DPPH: 2,2-diphenyl radical methods and ORAC: oxygen radical absorbance capacity.

Method	Standard	Equation	R^2	DL	QL	LR	CV%
Folin	Gallic acid	y = 0,0005x - 0,00367	0.9925	12.489 mg L^{-1}	41.633 mg L^{-1}	41.633 – 500 mg L^{-1}	3.28
TEAC	Trolox	y = 0,3104x + 0,1593	0.9958	0.048 mmol L^{-1}	0.162 mmol L^{-1}	0.162- 0.7 mmol L^{-1}	4.64
CUPRAC	Trolox	y = 3.0604x + 0.1344	0.9961	0.055 mmol L^{-1}	0.183 mmol L^{-1}	0.183 – 0.7 mmol L^{-1}	1.49
DPPH	Trolox	y = 0.5996x + 0.0139	0.9957	0.043 mmol L^{-1}	0.144 mmol L^{-1}	0.144 – 0.7 mmol L^{-1}	4.53
ORAC	Trolox	y = 0.6457x + 10.185	0.9905	2.170 umol L^{-1}	7.234 umol L^{-1}	7.234 – 80 umol L^{-1}	4.10
HPLC	Cyanidin-3-glucoside	y = 63289X + 3818.7	1	0.072 mg L^{-1}	0.241 mg L^{-1}	0.241 – 100 mg L^{-1}	1.22
	Quercetin	y=13318x + 1424.8	0.9999	0.102 mg L^{-1}	0.340 mg L^{-1}	0.340 – 100 mg L^{-1}	4.41
	Chlorogenic acid	y= 73284x + 6553.5	1	0.042 mg L^{-1}	0.140 mg L^{-1}	0.140 - 100 mg L^{-1}	0.46
	Citric acid	y= 1274.2x – 3763.9	0.9996	0.198 mg L^{-1}	0.661 mg L^{-1}	0.661 – 500 mg L^{-1}	1.36
	Catechin	y= 57083x + 3800.6	1	0.067 mg L^{-1}	0.224 mg L^{-1}	0.224 – 100 mg L^{-1}	0.11

Figure S1: A) *Rosa rubiginosa* and B) *Rosa canina* fruits.

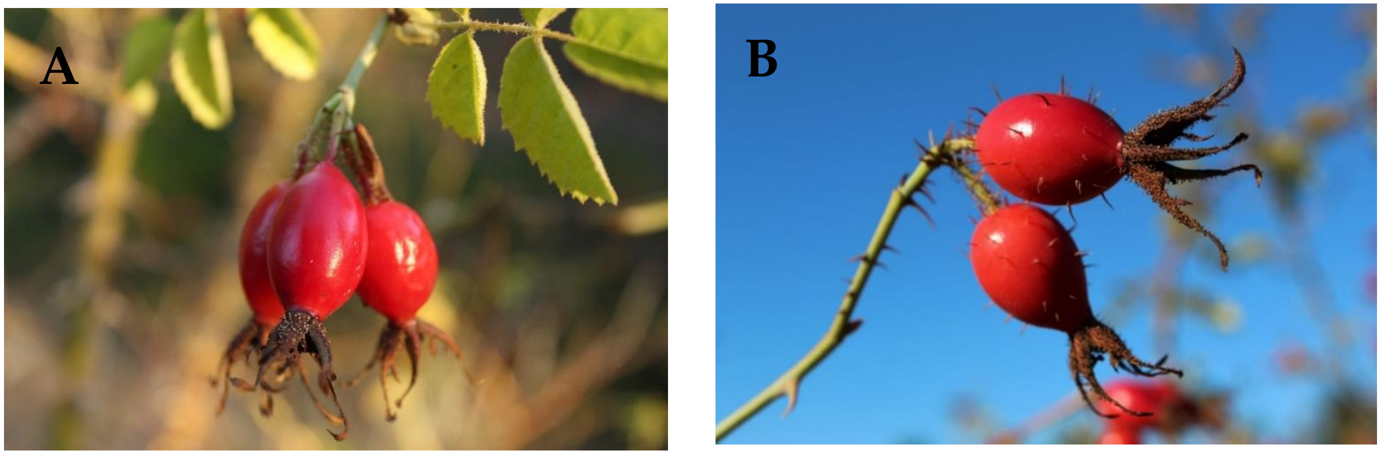

Supplement: Supplementary file 1 [file molecules-28-03544-s001.zip › molecules-2318503-supplementary.pdf]
